# Targeting the long non-coding RNA MIAT for the treatment of fibroids in an animal model

**DOI:** 10.1042/CS20240190

**Published:** 2024-06-07

**Authors:** Tsai-Der Chuang, Nhu Ton, Nathaly Manrique, Shawn Rysling, Omid Khorram

**Affiliations:** 1 Department of Obstetrics and Gynecology, Harbor-UCLA Medical Center, Torrance, CA, U.S.A.; 2 The Lundquist Institute for Biomedical Innovation, Torrance, CA, U.S.A.; 3 Department of Obstetrics and Gynecology, David Geffen School of Medicine at University of California, Los Angeles, Los Angeles, CA, U.S.A.

**Keywords:** Extracellular matrix, Fibroid, Lentivirus, MIAT, Xenografts

## Abstract

Our previous studies indicated that there is overexpression of MIAT in fibroids and MIAT is a sponge for the miR-29 family in these tumors. The objective of the present study was to determine if the knockdown of MIAT in fibroid xenografts will increase miR-29 levels and reduce the expression of genes targeted by this miRNA such as collagen and cell cycle regulatory proteins in a mouse model for fibroids. Ovariectomized CB-17 SCID/Beige mice bearing estrogen/progesterone pellets were implanted subcutaneously in the flank with equal weight of fibroid explants which had been transduced by lentivirus for either control (empty vector) or MIAT knockdown for four weeks (*n*=7). Knockdown of MIAT in fibroid xenografts resulted in a 30% reduction of tumor weight and a marked increase in miR-29a, -b, and -c levels in the xenografts. There was reduced cell proliferation and expression of cell cycle regulatory genes CCND1, CDK2, and E2F1 and no significant changes in apoptosis. The xenografts with MIAT knockdown expressed lower mRNA and protein levels of FN1, COL3A1, and TGF-β3, and total collagen protein. Targeting MIAT, which sponges the pro-fibrotic miR-29 family, is an effective therapy for fibroids by reducing cell proliferation and thereby, tumor growth and accumulation of ECM, which is a hallmark of these benign gynecologic tumors.

## Introduction

The focus of our laboratory has been to identify the mechanisms underlying the dysregulation of protein-coding genes in uterine fibroids, a benign gynecologic tumor affecting up to 80% of reproductive-age women, and the most common indication for hysterectomies performed worldwide. The role of long non-coding RNAs as a contributing factor to aberrant expression of protein-coding genes is largely unexplored. LncRNAs are greater than 200 nucleotides in length [1,2] and can modulate gene expression through several mechanisms including sponging of miRNAs and through epigenetic mechanisms such as altering chromatin structure and chromatin looping, and recruiting transcription factors or RNA polymerase 2 and release of transcription repressors [3–6]. In a recent study, we reported that several lncRNAs are aberrantly expressed in fibroids [7,8]. A few studies have addressed the functional role of lncRNAs in fibroid pathogenesis. Among these lncRNA, H19 was reported to be overexpressed in fibroids and regulated the expression of genes related to inflammation, ECM composition, and cell proliferation via epigenetic modification by TET3 (Tet methylcytosine dioxygenase 3) [9]. Our group reported that XIST was overexpressed in fibroids and acted as a ceRNA for miR-200c and miR-29c, lowering the levels of these miRNAs and up-regulation of their target genes including fibronectin and COL1A1 and COL3A1 [10]. Similarly, MIAT was also overexpressed in fibroids more so in tumors with MED12 mutation. In the present study, we demonstrated that MIAT targets the miR-29 family which was previously shown by us and others to be a key miRNA in fibroid pathogenesis [11–13] through its regulation of target genes involved in ECM composition such as collagen subtypes. In *in vitro* studies we reported that knockdown of MIAT by transient transfection of siRNA in LSMC spheroids resulted in up-regulation of miR-29, and down-regulation of its target genes including TGF-β3 (transforming growth factor β-3), collagen type III (COL3A1), and collagen type I (COL1A1). Based on these findings we hypothesized that downregulation of MIAT would result in up-regulation of miR-29 which in turn would result in down-regulation of target genes for this key miRNA including genes regulating ECM composition such as collagen and TGF-β3 and genes that regulate the cell cycle such as CDK2. We tested this hypothesis in a mouse model for fibroids which we have previously characterized [14,15].

## Materials and methods

### Fibroid specimens collection

Portions of intramural uterine fibroids (2–5 cm diameter) (*n*=7) were obtained from hysterectomies performed for symptomatic fibroids at the Harbor-UCLA Medical Center. Prior approval from the Institutional Review Board (18CR-31752-01R) at the Lundquist Institute was obtained. Tissues were obtained only from premenopausal patients not using hormonal medications 3 months prior to surgery. Informed consent was obtained from all the participants. Portions of the tumors were used for *in vivo* studies and the remainder, along with myometrium, was snap-frozen in liquid nitrogen for molecular analysis [16,17].

### Fibroid animal model

The protocol (31752-01) was approved by the IACUC at the Lundquist Institute at the Harbor-UCLA Medical Center. Female ovariectomized SCID/Beige mice (Charles River Laboratories, Hollister, CA, U.S.A.) 9-12 weeks old were implanted with pellets (Innovative Research of America, Sarasota, FL, U.S.A.) containing estradiol (0.075 mg/60 d release) and progesterone (75 mg/60 d release) as previously reported [15]. MIAT was knocked down by the lentivirus-based siRNA method using a lentivector (cat# 61891091) from Applied Biological Materials Inc. For lentivirus generation and explant transduction, 293T cells were transfected with lentivirus vectors along with package plasmids (pMD2.G and psPAX2), and supernatants were collected. A portion of fresh fibroid weighing 0.5 g was cut aseptically into 5–10 pieces using a razor blade and was transduced overnight by lentivirus with MOI of 10 for either control vector (cat# LV015-G) or siMIAT vector as mentioned above. Equal weights of 7 pairs of explants collected from 7 patients were then implanted subcutaneously in the flank of 14 mice, thus allowing comparison of each treated tumor to its own control. After one month all mice were anesthetized with Ketamine (100 mg/kg) plus Xylazine (10 mg/kg) injected intraperitoneally, and blood was obtained by cardiac puncture. Mice were then subjected to cervical dislocation. The xenografts were dissected free of surrounding tissue and then were weighed and frozen. Animals were weighed before implantation of xenografts and just prior to sacrifice. All surgical procedures and associated experiments with mice were performed in the C.W. Steers Biological Resources Center (BRC) animal care facility at the Lundquist Institute.

### Blood chemistry panel

The plasma chemistry was analyzed by the VetScan VS2 chemistry analyzer (Abaxis, Union City, CA, U.S.A.) (Comprehensive Diagnostic Profile Rotor (#500-1038, Abaxis, Union City, CA, U.S.A.) according to the manufacturer’s protocol as previously described [14,15].

### Quantitative RT-PCR

Quantitative RT-PCR was done as previously described [18]. FBXW2 (F-box and WD repeat domain containing 2) was used for normalization [19]. The reactions were run in triplicate and were used for determining the relative mRNA expression. The primer sequences used were as follows: COL3A1 (sense, 5′- ATTATTTTGGCACAACAGGAAGCT-3′; antisense, 5′- TCCGCATAGGACTGACCAAGAT-3′); FN1 (sense, 5′- ACCGAAATCACAGCCAGTAG-3′; antisense, 5′-CCTCCTCACTCAGCTCATATTC-3′); TGF-β3 (sense 5′-CGGGCTTTGGACACCAATTA-3′, antisense 5′-GGGCGCACACAGCAGTTC-3′); IL8 (sense, 5′-CTTGGCAGCCTTCCTGATTT-3′; antisense, 5′-TTCTTTAGCACTCCTTGGCAAAA-3′); CCND1 (sense, 5′- GCCCTCTGTGCCACAGATGT-3′; antisense, 5′-CCCCGCTGCCACCAT-3′); CDK2 (sense, 5′-TTCCCCTCATCAAGAGCTATCTGT-3′; antisense, 5′-ACCCGATGAGAATGGCAGAA-3′); E2F1 (sense, 5′-GGACTCTTCGGAGAACTTTCAGATC-3′; antisense, 5′- GGGCACAGGAAAACATCGA-3′); and FBXW2 (sense, 5′- CCTCGTCTCTAAACAGTGGAATAA-3′; antisense, 5′- GCGTCCTGAACAGAATCATCTA-3′).

### Immunoblotting

Protein isolated from the xenografts was subjected to immunoblotting as previously described with primary antibodies against COL3A1 (dilution 1:3000, 22734-1-AP, Proteintech Group, Inc, Rosemont, IL, U.S.A.), FN1 (dilution 1:3000, 15613-1-AP, Proteintech Group, Inc, Rosemont, IL, U.S.A.), and TGF-β3 (dilution 1:1500, 18942-1-AP, Proteintech Group, Inc, Rosemont, IL, U.S.A.) [20]. The specific protein band densities were assessed through the ImageJ program (http://imagej.nih.gov/ij/), normalized to a band of approximately 63 kDa obtained from Ponceau S staining on the membrane. The results were presented as means ± SEM, represented as a ratio relative to the control group, designated as 1.

### Enzyme-linked immunosorbent assay

The total collagen content in xenografts was determined in duplicate using the QuickZyme Total Collagen Assay Kit (QuickZyme Biosciences, Leiden, Netherlands) according to the manufacturer’s instructions as previously described [15].

### Immunohistochemistry

Tissues were fixed in 4% paraformaldehyde and embedded in paraffin. Five-micron sections were cut and mounted on glass slides. The Masson’s trichrome stain (HT15-1KT, Sigma-Aldrich) and the immunohistochemistry were performed as previously described [14,15]. The primary antibodies used in this study are rabbit anti-Ki67 (dilution 1:250, 27309-1-AP, Proteintech Group, Inc, Rosemont, IL, U.S.A.), rabbit anti-cleaved caspase-3 (dilution 1:50, #9664, Cell Signaling Technology, Danvers, MA, U.S.A.), rabbit anti-COL3A1 (dilution 1:1000, 22734-1-AP, Proteintech Group, Inc, Rosemont, IL, U.S.A.), and mouse anti-E2F1 (dilution 1:50, sc-251, Santa Cruz Biotechnology, Inc., Dallas, TX, U.S.A.).

### Statistics and power analysis

Data was analyzed by GraphPad Prism 10 software (Graph-Pad, San Diego, CA, U.S.A.). The normality of data was determined by the Kolmogrove–smirnoff test. We used a non-parametric test for data analysis because the data were not normally distributed. Comparisons between two groups were assessed using the Wilcoxon matched pairs signed rank test, while correlation analysis was conducted using the Spearman test. Statistical significance was set at *P*<0.05.

## Results

**Figure 1:**
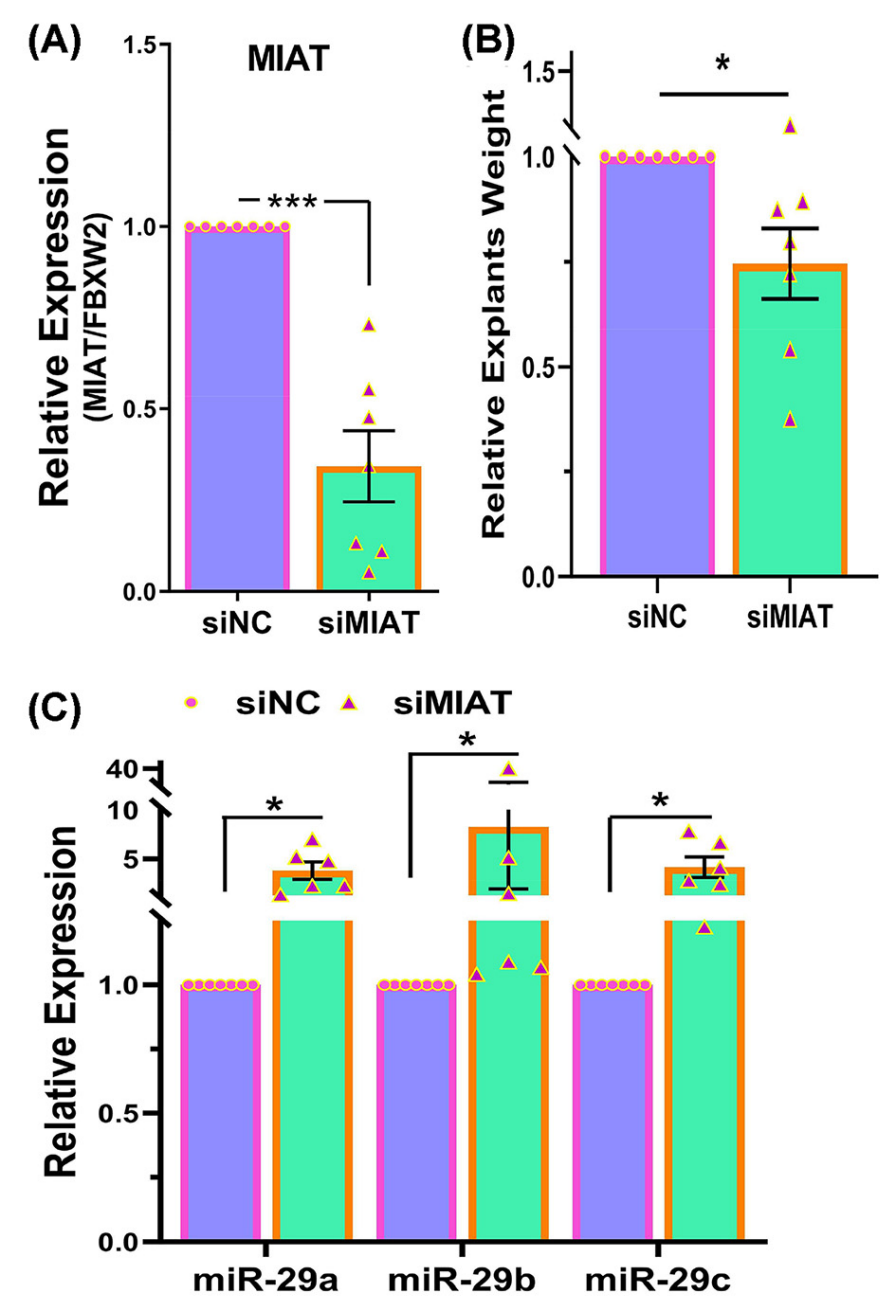
The impact of MIAT knockdown on tumor weight and miR-29 family levels in fibroid xenografts. Fresh fibroids explants transduced by lentivirus for either control (siNC) or MIAT (siMIAT) knockdown (*n*=7). (**A**) Relative mRNA expression of MIAT. (**B**) The weight of tumor explants was determined after 4 weeks. (**C**) Relative levels of miR-29a, miR-29b, and miR-29c. The results are displayed as the mean ± SEM of separate experiments, with p values specified on the respective lines. *P<0.05, ***P<0.001.

The knockdown of MIAT in fibroid xenografts was well tolerated by mice as indicated by the blood chemistry panel shown in Table 1. There were no significant differences in body weight gain between the two groups of mice over the 4-week course of the experiment (siNC vs. siMIAT: 2.739 g ± 0.183 vs. 2.576 g ± 0.192). As demonstrated in Figure 1A the knockdown of MIAT with siRNA was highly effective in reducing the expression of MIAT by 65% 4-week post-transduction. Within this short period the weight of fibroid tumors decreased by 30% in xenografts in which MIAT was knocked down (Figure 1B). As predicted the suppression of MIAT which is a sponge or ceRNA for the miR-29 family led to an increased level of miR-29 family in the xenografts (Figure 1C).

**Table 1:** Chemistry panel of *in vivo* knocking down of MIAT in mice

Chemistry Panel Marker	siNC (mean ± SEM)	siMIAT (mean ± SEM)	*P*-value
General metabolism			
Glucose (mg/dl)	226.4 ± 21.77	238.6 ± 20.4	0.6933
Kidney function			
BUN (mg/dl)	22.6 ± 2.25	26 ± 2.7	0.3618
Creatinine (mg/dl)	0.3 ± 0.03	0.2 ± 0.02	0.195
Electrolytes			
Sodium (mEq/l)	157.0 ± 6.23	152.8 ± 2.58	0.5506
Phosphorus (mg/dl)	10.0 ± 0.87	10.9 ± 0.34	0.3438
Liver function			
Alkaline phosphatase (U/l)	114.4 ± 13.37	116.8 ± 6.135	0.8744
Albumin (g/dl)	3.5 ± 0.25	3.3 ± 0.09	0.3869
SGPT (ALT) (U/l)	78.2 ± 30.65	80 ± 21.65	0.9629
Total protein (g/dl)	4.6 ± 0.26	4.3 ± 0.0	0.3666
Globulin (g/dl)	1.1 ± 0.13	1.0 ± 0.07	0.7942
Total bilirubin (mg/dl)	0.2 ± 0.0	0.2 ± 0.0	0.172
Pancreas function			
Amylase (U/l)	736.8 ± 64.41	674.4 ± 46.45	0.4546

**Figure 2:**
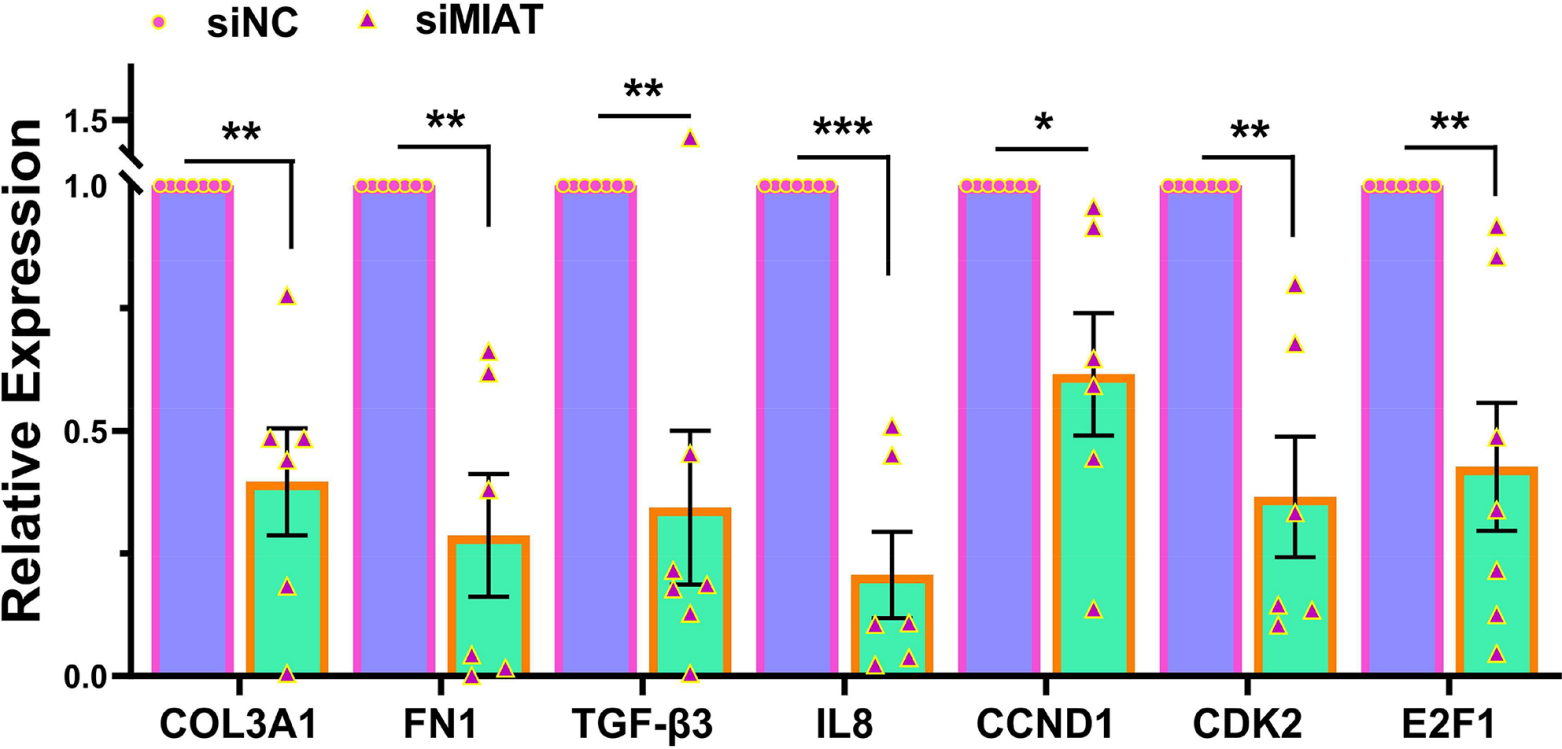
The effect of MIAT knockdown on fibroid-associated gene expression levels Relative mRNA expression of COL3A1, FN1, TGF-β3, IL8, CCND1, CDK2, and E2F1 in xenografts implanted subcutaneously in ovariectomized CB-17 SCID/Beige mice (*n*=7) after 4 weeks of MIAT knockdown by lentivirus.

**Figure 3:**
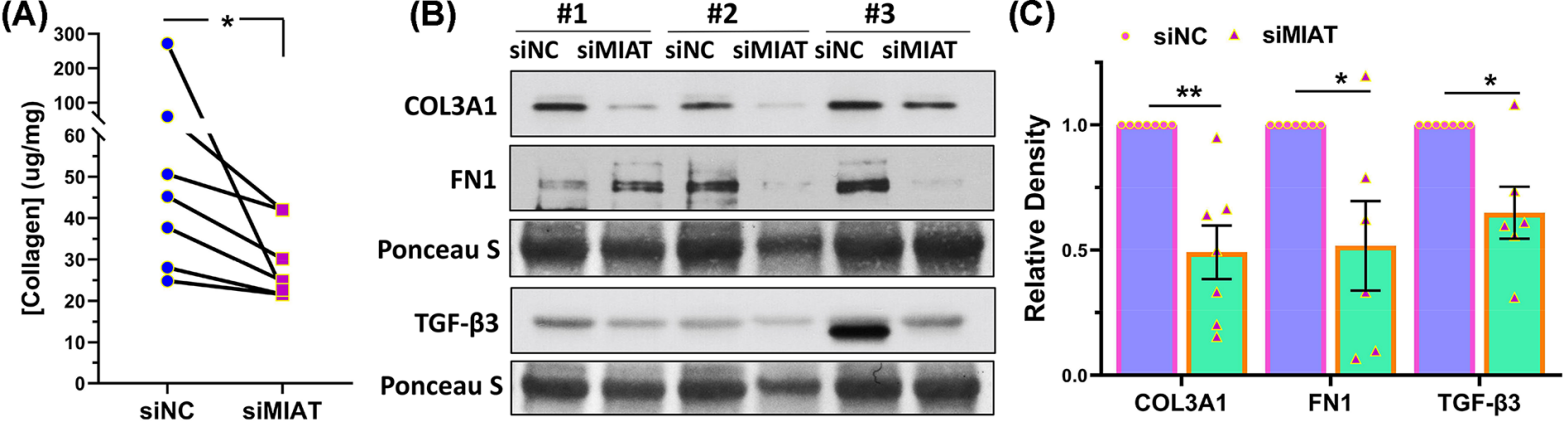
The impact of MIAT knockdown on protein expression levels associated with fibroids (**A**) Total collagen levels as determined by enzyme-linked immunosorbent assay in seven xenografts. (**B**) Representative Western blot analysis of COL3A1, FN1, and TGF-β3 with bar graphs (**C**) showing their relative band densities in the xenografts (*n*=7). The results are displayed as the mean ± SEM from distinct experiments, with *P-*values specified at the corresponding line. *P<0.05, **P<0.01.

**Figure 4:**
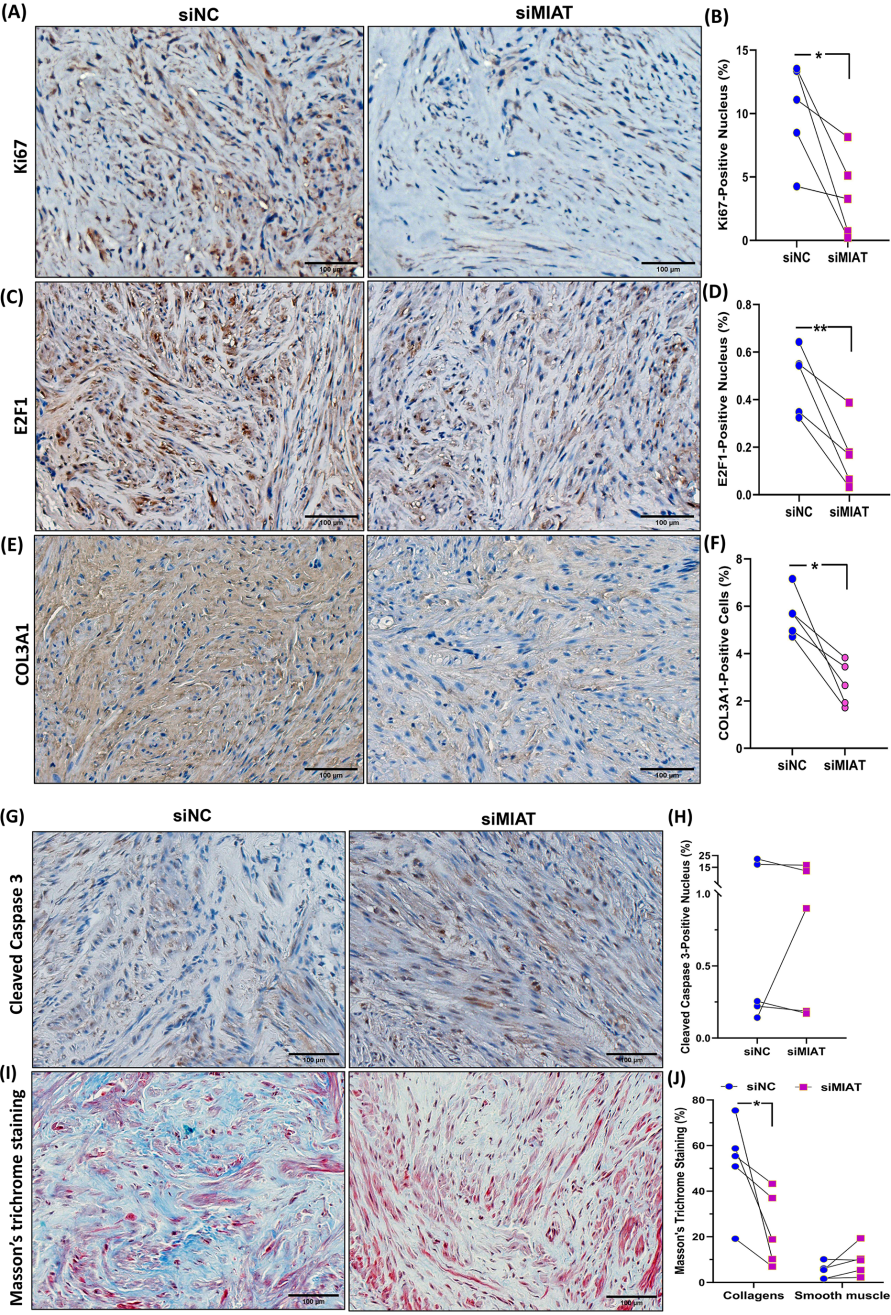
The effect of MIAT knockdown on fibroid-associated protein expression as shown by immunohistochemistry Representative immunohistochemically stained images of fibroid xenografts from control (siNC) or MIAT knockdown treatment group (*n*=5), and image analysis by Halo software were shown for Ki67 (**A,B**), E2F1 (**C,D**), COL3A1 (**E,F**), and Cleaved Caspase 3 (**G,H**). (**I**) Representative histologic staining of fibroid xenografts by Masson’s trichrome staining from control (siNC) or MIAT knockdown treatment group. Collagen fibers are represented by the blue color, while smooth muscle cells are indicated in red. (J) Shows the quantification of staining intensity by Halo software. The results are displayed as mean ± SEM, with *P* values specified at the corresponding line. *P<0.05.

**Figure 5:**
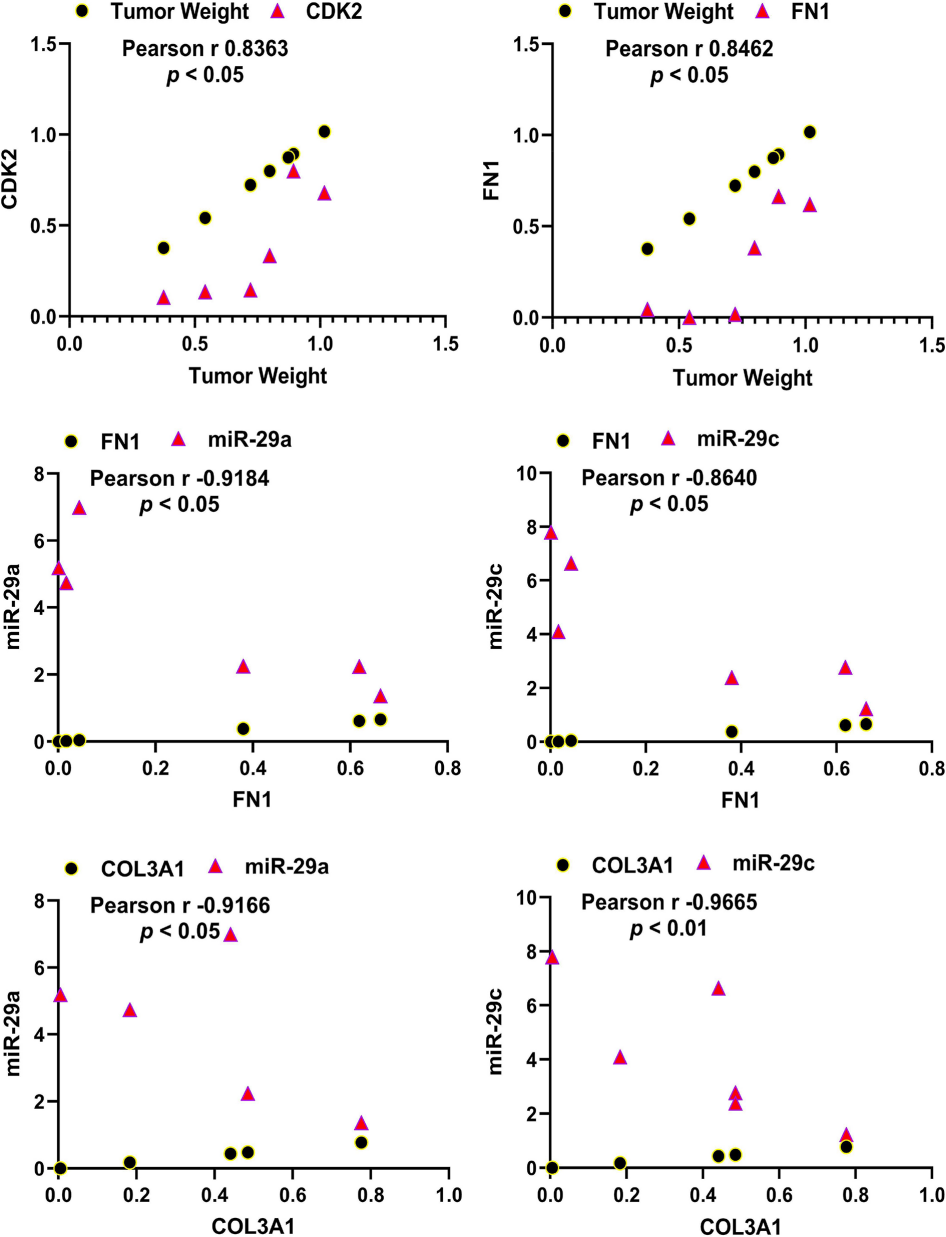
The impact of MIAT knockdown on gene correlations in fibroid xenografts Correlation analysis between tumor weight and mRNA levels of CDK2 and FN1 in fold change (siMIAT/siNC), and the levels of miR-29a and miR-29c with mRNA expression of FN1 and COL3A1 in the xenografts (*n*=7).

The knockdown of MIAT resulted in decreased mRNA expression of several genes including three key components of the ECM namely FN1, COL3A1, and TGF-β3, key cell cycle regulatory genes (CDK2, E2F1, CCND1), and the inflammatory cytokine (IL8) (Figure 2). Of these genes COL3A1, TGF-β3 and CDK2 are targets of the miR-29 family [11,21,22]. The knockdown of MIAT also reduced the expression of total collagen species as determined by ELISA (Figure 3A). Western blot analysis indicated a reduction in COL3A1, FN1, and TGF-β3 protein levels in the xenografts with MIAT knockdown (Figure 3B,C). Immunohistochemical and image analysis of the xenografts indicated that knockdown of MIAT reduced cell proliferation and collagen levels as indicated by reduced Ki67 (Figure 4A,B), E2F1 (Figure 4C,D) and COL3A1 (Figure 4E,F) staining and no significant changes in apoptosis as indicated by cleaved caspase staining (Figure 4G,H). Histologic analysis using Masson’s trichrome staining indicated reduced collagen and proteoglycan abundance and no changes in smooth muscle (Figure 4I,J). We detected a significant positive correlation between the tumor weight and CDK2 and FN1 mRNA expression and a negative correlation between miR-29a and miR-29c and mRNA levels of FN1 and COL3A1 (Figure 5).

## Discussion

The results of this study indicate that knockdown of MIAT in fibroid xenografts effectively reduced MIAT levels by 65% causing a 30% reduction of tumor weight within a relatively short period of time. This reduction in MIAT levels led to markedly increased levels of the miR-29 family for which MIAT is a ceRNA. There was a significant reduction in the expression of several genes targeted by miR-29 in the xenografts, including COL3A1, CDK2, and TGF-β3. The xenografts with MIAT knockdown showed reduced cell proliferation and no changes in cellular apoptosis. Our results indicate that modulation of lncRNA levels, which target key miRNAs that play a role in fibroid pathogenesis, such as the miR-29 family, could be an effective novel therapeutic approach for fibroids.

Several studies have provided evidence for MIAT as a tumor-promoting lncRNA in multiple types of cancer through its effects to regulate gene expression at the transcriptional or post-transcriptional levels [23,24]. At the transcriptional, its effect is exerted in the nucleus through interaction with nuclear factors such as DNMTs [25], and at the post-transcriptional levels, this is mediated through its interaction with cell-specific miRNAs [24]. In both *in vitro* studies and *in vivo* studies knockdown of MIAT has been reported to repress cell proliferation and reduce tumor growth [23,24] similar to our findings in fibroids which are benign tumors. Many of these studies also showed the involvement of the same cell cycle regulatory proteins we found to be inhibited following MIAT knockdown including CCND1 [26], and CDK2 [27]. To our knowledge other than our study there are no other reports showing an effect of MIAT knockdown on E2F1 expression. The down-regulated cell cycle regulatory proteins (CDK2, CCND1, E2F1) in this study were previously demonstrated to be up-regulated in fibroids [21]. In contrast to many studies in malignant tumors where MIAT knockdown induced apoptosis [28,29] we did not detect significant differences in cellular apoptosis, indicating cell specificity for MIAT action and probable differences in its mechanism of action in malignant versus benign tumors. The effects of MIAT in malignant tumors on cell proliferation and apoptosis are mediated by many different miRNAs depending on the cancer type [23,24]. In several types of cancer, the tumorigenic effects of MIAT were mediated through its interaction with miR-29 family [30–32]. In this study, we focused on the detection of miR-29 and its target genes including ECM, cell cycle, and inflammatory genes based on our previous studies showing down-regulation of miR-29 and up-regulation of MIAT in fibroids and demonstration of miR-29 as being a target of MIAT in fibroids [20]. However, we cannot preclude the involvement of other miRNAs for which MIAT is a ceRNA for the observed effects.

MIAT also plays a significant role in fibrotic disorders [33–37] and depending on the tissue and cell type it sponges different miRNAs. The link between MIAT and miR-29 has been reported in several fibrotic disorders. In a rat model of pulmonary hypertension MIAT stimulated oxidative stress by sponging miR-29a-5p and inhibited the Nrf2 pathway [38]. Prenatal exposure to nicotine induced the expression of MIAT in the heart of offspring pups, and this resulted in the sequestration of the miR-29 family and up-regulation of collagens [39]. In diabetic retinopathy, overexpression of MIAT leads to sequestration of miR-29b and resultant increased expression of Sp1 expression, which is a target gene of the miR-29 family [40]. The same inverse relationship between MIAT and miR-29a was reported in a rat model of kidney injury [41]. MIAT was also reported to regulate fibrosis in hypertrophic cardiomyopathy through miR-29 [34]. The down-regulation of ECM genes (COL3A1, FN1), cell cycle regulatory proteins (CCND1, CDK2, E2F1), the inflammatory marker (IL8), and the negative correlation between miR-29 and COL3A1 and FN1 in MIAT knocked down xenografts supports the role of miR-29 as at least one of the miRNAs mediating these effects.

The current medical therapies for fibroids have significant limitations secondary to side effects from hypoestrogenism or endometrial effects thus limiting their use for extended periods [42]. Alternative therapies that do not inhibit estrogen production causing hypoestrogenic side effects are urgently needed for treatment of fibroids. We have previously provided preclinical *in vivo* data for two therapies in a mouse model, including treatment with the anti-inflammatory drug tranilast [14] and more recently using a drug that inhibits the activity of the tryptophan catabolizing enzyme TDO2 [15] which is markedly overexpressed in fibroids [43,44]. Because of their localized presence and slow growth treatment of fibroids by gene therapy approaches have attracted some attention with some promising results. Most of these studies have been *in vitro* with a limited number of *in vivo* studies where viruses have been used for transduction of dominant negative estrogen receptor, suicide genes, anti-angiogenic genes and by mutation compensation [45,46]. Our data indicate that targeting lncRNAs which sponge key aberrantly expressed miRNA in fibroids either by viral transfection approaches or drugs that could alter their expression could be another approach for fibroid treatment.

In summary, the data presented here provides evidence for the important role of the pro-tumorigenic and fibrotic lncRNA MIAT in fibroid pathogenesis. Knockdown of MIAT in fibroid xenografts reduced tumor weight by inhibiting cell proliferation and down-regulation of key cell cycle regulatory proteins CDK2, CCND1, and E2F1. The knockdown of MIAT also reduced its pro-fibrotic effects by inhibiting the expression of collagens, TGF-β3 and FN1. These effects of MIAT are mediated at least in part through sponging of the miR-29 family as evidenced by marked increased levels of miR-29a, b, and c in the xenografts with MIAT knockdown. Gene therapy approaches targeting key dysregulated lncRNAs such as MIAT could be a novel approach for the treatment of fibroids.

## Clinical perspectives

- Background as to why the study was undertaken. Our previous work showed a lncRNA MIAT functions as a competing endogenous RNA for miR-29 family and is involved in extracellular matrix deposition in fibroids.
- A brief summary of the results. Xenografts from the MIAT knockdown group showed a 65% inhibition of MIAT levels and 30% reduction in tumor weight after 4 weeks of treatment along with a significant decrease in staining for Ki67 and E2F1, indicating reduced cells proliferation. Knockdown of MIAT significantly reduced Masson’s trichrome staining of the xenografts and COL3A1 staining, indicative of reduced abundance of collagen and proteoglycans. There was a significantly increased level of miR-29 family in the xenografts confirming our previous *in vitro* data, and inhibition of miR-29 family targets including COL3A1, TGF-β3 and CDK2 in the xenografts from the MIAT knockdown group.
- The potential significance of the results to human health and disease. Our results underscore the physiological significance of MIAT in fibroid pathogenesis and a potential gene therapy approach targeting this lncRNA to reduce fibroid cell proliferation and ECM accumulation.

## Data Availability

Raw data were generated at The Lundquist Institute. Derived data supporting the findings of this study are available from the corresponding author O.K. on request.

## Abbreviations

COL1A1: collagen type I
COL3A1: collagen type III
DNMT: DNA methyltransferases
ECM: Extracellular matrix
MIAT: Myocardial infarction-associated transcript
TDO2: Tryptophan 2,3-dioxygenase
TET3: Tet methylcytosine dioxygenase 3
TGF-β3: transforming growth factor β-3
